# A finite volume-based model for the hydrothermal behavior of soil under freeze–thaw cycles

**DOI:** 10.1371/journal.pone.0252680

**Published:** 2021-06-03

**Authors:** Tianfei Hu, Tengfei Wang

**Affiliations:** 1 State Key Laboratory of Mechanical Behavior and System Safety of Traffic Engineering Structures, Shijiazhuang Tiedao University, Shijiazhuang, China; 2 School of Civil Engineering, Shijiazhuang Tiedao University, Shijiazhuang, China; 3 MOE Key Laboratory of High-Speed Railway Engineering, Southwest Jiaotong University, Chengdu, China; 4 School of Civil Engineering, Southwest Jiaotong University, Chengdu, China; Texas A&M University System, QATAR

## Abstract

Freeze–thaw cycles in soil are driven by water migration, phase transitions, and heat transfer, which themselves are closely coupled variables in the natural environment. To simulate this complex periglacial process at different time and length scales, a multi-physics model was established by solving sets of equations describing fluid flow and heat transfer, and a dynamic equilibrium equation for phase changes in moisture. This model considers the effects of water–ice phase changes on the hydraulic and thermal properties of soil and the effect of latent heat during phase transition. These equations were then discretized by using the finite volume method and solved using iteration. The open-source software OpenFOAM was used to generate computational code for simulation of coupled heat and fluid transport during freezing and thawing of soil. A set of laboratory freezing tests considering two thermal boundary conditions were carried out, of which the results were obtained to verify the proposed model. In general, the numerical solutions agree well with the measured data. A railway embankment problem, incorporating soil hydrothermal behavior in response to seasonal variations in surface temperature, was finally solved with the finite volume-based approach, indicating the algorithm’s robustness and flexibility.

## Introduction

Unsaturated soil at or near to the ground surface may experience frequent freeze–thaw cycles driven by seasonal climate change. The associated change of soil temperature, fluid flow, and formation of ice all affect the long-term performance of infrastructure, posing problems for engineers [1–3]. As such, being able to predict fluid flow patterns and temperature gradients in soil is of great significance for geotechnical projects in cold regions.

The successive freezing and thawing of soil mass are essentially a heat and mass transfer process, which is governed by thermal conduction, the migration, and phase transformation of water in the subsurface environment. The migration and freezing of water are closely coupled processes, and jointly influence the temperature field and the rate of heat transfer within soil. Two types of mathematical models have previously been developed to describe this coupling strategy: mechanistic models based on the viscous flow of liquid water in porous media and the thermal balance of soil [4], and thermodynamic models that apply principles of irreversible thermodynamics for water and heat fluxes within soil [5, 6]. A variety of numerical codes use mechanistic models when applied to geotechnical engineering problems, which is a technique that we adopt in this study.

Many previous studies have laid a solid foundation for the parameterization of complex numerical models. Harlan [7] presented the first finite difference solution to a one-dimensional hydrothermal problem regarding the freezing and thawing of a homogeneous, porous medium. Subsequently, Newman et al. [8] presented an approach for predicting heat and mass transfer in freezing, unsaturated soil. In their work, characteristics for soil–ice and soil–water mixtures were used to combine heat and mass transfer relationships into a single equation for freezing (or frozen) soils. Hansson et al. [9, 10] presented a robust, energy- and mass-conservative solution to a coupled heat transport and variably saturated water flow, which was designed to apply to a temperature range across 0°C. In addition, several unusual water-migration phenomena that occur during soil freezing have also been reported and studied comprehensively, such as the frozen fringe and its suction effect [11, 12], the canopy effect [13, 14], and the excess pore-water pressure effect [15].

Based on these investigations, several codes have been developed for modeling the freeze–thaw cycles, including the HYDRUS model [16], the FROST model [17], the SHAW model [18], the SOIL model [19], and the VAPOR model [20]. These codes are sometimes unsuitable for addressing more complex problems, as they are limited to 1D or 2D problems. In addition, because of their geometric adaptability, they can only be applied to structured grids, and these codes do not support parallel computing, which limits the size of problems to be investigated. In general, simulation capabilities accounting for complex geometries and high-resolution problems are essentially limited.

In light of this, we have adopted an unstructured mesh-based code to addresses these gaps in modeling capabilities, namely the finite element method (FVM). In the FVM, volume integrals in a partial differential equation that contain a divergence term are converted to surface integrals, using the divergence theorem. These terms are then evaluated as fluxes at the surfaces of each finite volume. In contrast to the finite element method and finite difference method, the FVM evaluates exact expressions for the average value of the solution over volume, and uses this data to construct approximations of the solution within cells. The FVM-based approach shows notable advantages of automatic matrix construction, solution capabilities for partial differential equations, and ease of implementation in some open-source platforms, such as OpenFOAM [21]. The FVM code developed in this study was validated by laboratory freezing test data, and further tested with seasonal simulation of a railway embankment case involving heat transport and water flow in response to seasonal variations in surface temperature.

The remainder of paper is organized as follows: Section 2 presents the fundamentals for the coupled heat transfer and water migration process in the soil domain; Section 3 describes the finite volume method and developing strategy for the coupled model; Section 4 presents the validation and application of the FVM code; Finally, conclusions and some proposals for future investigations are given in Section 5.

## Coupled hydrothermal model

### Heat transfer

Transitions between unfrozen, frozen, and thawing soil are necessarily accompanied by conductive heat transfer and changes in the physical state of H_2_O. According to heat transfer theory, the control equation for unsteady heat transfer can be formulated in terms of the latent heat of phase changes being either a heat source or sink [22], given as

$$\frac{\partial CT}{\partial t}=\nabla \cdot \lambda_{\text{eff}}\nabla T+L\rho_{i}\frac{\partial \theta_{i}}{\partial t}$$ (1)

where *C* is the volumetric heat capacity of the soil, J∙m^−3^∙K^−1^; *T* is the soil temperature, K; *λ* is the soil thermal conductivity, W∙m^−1^∙K^−1^; *L* is the latent heat released as water transforms into ice or vice versa, J∙kg^−1^; *ρ*_i_ is the ice density, kg∙m^−3^; and *θ*_i_ is the volumetric ice content.

During the freezing or thawing of soil, pore water is redistributed and *θ*_*i*_ changes simultaneously. The physical and mechanical properties of the soil also change due to variations in soil–water–ice proportions. The volumetric heat capacity of soil can be expressed as the sum of the volumetric heat capacity of the solid matrix, liquid water, and ice phases multiplied by their volumetric fractions, expressed as [23]

$$C=\theta_{s}C_{s}+\theta_{i}C_{i}+\theta_{u}C_{u}$$ (2)

where *θ*_*s*_ is the volumetric soil matrix content, *C*_*s*_ is the volumetric heat capacity of soil matrix, J∙m^−3^∙K^−1^; *C*_*i*_ is the volumetric heat capacity of ice, J∙m^−3^∙K^−1^; *θ*_*u*_ is the volumetric liquid water content, and *C*_*u*_ is the volumetric heat capacity of liquid water, J∙m^−3^∙K^−1^.

The effective thermal conductivity of soil depends on the thermal conductivity of all its components (i.e., soil matrix, liquid water, and ice), their volumetric fractions, and the spatial distribution of each phase. Thermal conductivity is generally obtained by a logarithmic law [23] from the physical properties of the soil matrix, ice, and water contents as follows

$$\lambda_{\text{eff}}=\lambda_{s}{}^{\theta_{s}}\lambda_{i}{}^{\theta_{i}}\lambda_{u}{}^{\theta_{u}}$$ (3)

where *λ*_*s*_ is the thermal conductivity of the soil matrix, W∙m^−1^∙K^−1^; *λ*_*i*_ is the thermal conductivity of ice, W∙m^−1^∙K^−1^; and *λ*_*u*_ is the thermal conductivity of water, W∙m^−1^∙K^−1^.

Soil moisture consists of liquid water located in pores or fractures within the soil, and ice located around the grain surface. Because of the difference in densities of ice and water, the volumetric moisture content of frozen soil is calculated by

$$\theta =\theta_{u}+\frac{\rho_{i}}{\rho_{w}}\theta_{i}$$ (4)

where *ρ*_*w*_ is the density of liquid water, kg∙m^−3^.

### Moisture migration

The water migration model is derived from the application of Darcy’s law to moisture movement in a permeable media. The migration of liquid water in soil at temperatures across 0°C is described by the modified Richards equation [23]

$$\frac{\partial \theta_{u}}{\partial t}+\frac{\rho_{i}}{\rho_{w}}\cdot \frac{\partial \theta_{i}}{\partial t}=\nabla [D(\theta_{u})\nabla \theta_{u}+K_{g}(\theta_{u})]$$ (5)

where *D*(*θ*_*u*_) is the soil water diffusivity, m^2^∙s^−1^; *K*(*θ*_*u*_) is the hydraulic conductivity of unsaturated frozen soil, m∙s^−1^.

The right first term in Eq (5) accounts for the fluid flow due to water concentration gradients, while the right second term represents the fluid flow by gravity. The water diffusivity in soil is then calculated by [9, 10]:

$$D(\theta_{u})=\frac{K(\theta_{u})}{C(\theta_{u})}\cdot I(\theta_{i})$$ (6)

where *C*(*θ*_*u*_) is the specific water capacity, m^−1^, *I*(*θ*_*i*_) represents the blocking effect of ice on water migration in frozen soil [9, 10], calculated by

$$I(\theta_{i})=10^{-10\theta_{i}}$$ (7)

In this study, the hydraulic properties are described by [24, 25]

$$K(\theta_{u})=K_{s}\cdot S^{l}{\left(1-{\left(1-S^{1/m}\right)}^{m}\right)}^{2}$$ (8)

$$C(\theta_{u})=a_{0}m/(1-m)\cdot S^{1/m}{\left(1-S^{1/m}\right)}^{m}$$ (9)

where *S* is the relative saturation of the frozen soil; *a*_*0*_, *l*, and *m* are empirical parameters; *K*_*s*_ is the saturated hydraulic conductivity, m∙s^−1^. The normalized saturation is given as [25]

$$S=\frac{\theta_{u}-\theta_{r}}{\theta_{s}-\theta_{r}}$$ (10)

where the *θ*_*r*_ is the residual volumetric water content, and *θ*_*s*_ is the volumetric water content of saturated soil.

### Coupling strategy

In the heat transfer and water migration models, there are three unknown variables, namely temperature, ice content, and liquid water content. An additional equation is required for a closed-form solution. In freezing soil, the unfrozen water content is seen as a function of temperature, generally determined from soil freezing characteristic curve [8]. If liquid water and the sub-zero temperature of frozen soil are in a dynamic equilibrium state, the relationship between liquid water contents and below-freezing temperatures is given by [26]

$$\frac{w_{0}}{w_{u}}={\left(\frac{T}{T_{f}}\right)}^{B},T<T_{f}$$ (11)

where *w*_*0*_ is the initial water content of unfrozen soil, *w*_*u*_ is the liquid water content of frozen soil, *T*_*f*_ is the freezing point, K, and *B* is a parameter related to the salt content and soil type, which can be determined by either using the one-point method [26] or by adopting typical values for silty sand (0.58) and sandy gravels (0.65). This relationship between liquid water contents and sub-zero temperatures can be represented as

w0wu=ρθiiρwθu+1 (12)

Eqs (11), (12) and (4) are combined to describe a relationship between the ice content, liquid water content, and temperature, expressed as

$$\theta_{i}=\{\begin{array}{ll} \frac{\rho_{\text{w}}}{\rho_{i}}\theta_{u}\cdot ({\left(\frac{T}{T_{f}}\right)}^{B}-1) & \left(T<T_{f}\right)\\ 0 & \left(T\geq T_{f}\right) \end{array}$$ (13)

Combining Eqs (1), (5) and (13) leads to the framework of a hydrothermal model. With proper boundary conditions, the temperature (*T*), volumetric liquid water content (*θ*_*u*_), and volumetric ice content (*θ*_*i*_) of a soil can be calculated. However, since analytical solutions to these equations are only available in special cases, numerical techniques must be employed to solve them.

## Numerical solution

Equations for heat transfer and water flow are both nonlinear. In this study, the Picard iteration method [27] is adopted to linearize both equations by allowing the nonlinearities to lag by one step behind. More specifically, both heat transfer and water flow equations are mathematically coupled through their phase change component, meaning that a mutual solution must be generated by iteration. In this section, we introduce a calculation method to address the coupling issue and the finite volume-based procedure for the subsequent simulation.

### Solution strategy

Eq (14) is used to eliminate the ice content variable in the definitions of heat and mass transfer, leaving both with only two undetermined variables.

$$\theta_{i}=r\theta_{u}$$ (14)

where the parameter *r* is expressed as

$$r=\{\begin{array}{ll} 1.1({\left(\frac{T}{T_{f}}\right)}^{B}-1) & \left(T<T_{f}\right)\\ 0 & \left(T\geq T_{f}\right) \end{array}$$ (15)

Then, the time derivative of *θ*_*i*_ can be rewritten as

$$\frac{\partial \theta_{i}}{\partial t}=\frac{\partial (r\theta_{u})}{\partial t}=\theta_{u}\frac{\partial r}{\partial t}+r\frac{\partial \theta_{u}}{\partial t}$$ (16)

Substituting Eq (16) into Eq (5) produces

$$(1+r\frac{\rho_{i}}{\rho_{w}})\frac{\partial \theta_{u}}{\partial t}=\nabla [D(\theta_{u})\nabla \theta_{u}+K_{g}(\theta_{u})]+\frac{\rho_{i}}{\rho_{w}}\frac{\partial r}{\partial t}\theta_{u}$$ (17)

In numerical calculations, the iterative process for each time step is given as:

1. The temperature *T*_*i*_^*k+1*^ at the end of the predicted time step *T*_*i*_^*k*^ is approximated by the temperature at the beginning of this step. The *i*^*k+1*^ component of the volumetric ice content (*θ*_*i*_) and liquid water content (*θ*_*u*_)_*i*_^*k+1*^ term at the end of this period is obtained by the dynamic equilibrium defined in Eq (13).
2. The (*θ*_*i*_)_*i*_^*k+1*^ expression obtained by the water diffusion coefficient *D*_*i*_^*k*^ and the result produced in Step (1) allows Eq (5) to be solved, and so the (*θ*_*u*_)_*i*_^*k+1*^ of each node at the end of this step is obtained. Furthermore, Eq (14) is used with this expression for (*θ*_*i*_)_*i*_^*k+1*^ to solve for *T*_*i*_^*k+1*^.
3. The (*θ*_*i*_)_*i*_^*k+1*^ expression obtained by *C*_*i*_^*k*^, *λ*_*i*_^*k*^, and the results of step (2) allows the temperature control Eq (1) to be solved, and the real *T*_*i*_^*k+1*^ of each node at the end of the period can be determined. This is both the correction value of this iteration step and the forecast value of the next iteration step.

The calculation is continued until the difference between unfrozen water content and temperature from two consecutive cycles satisfies the predefined tolerance.

**Fig 1:**
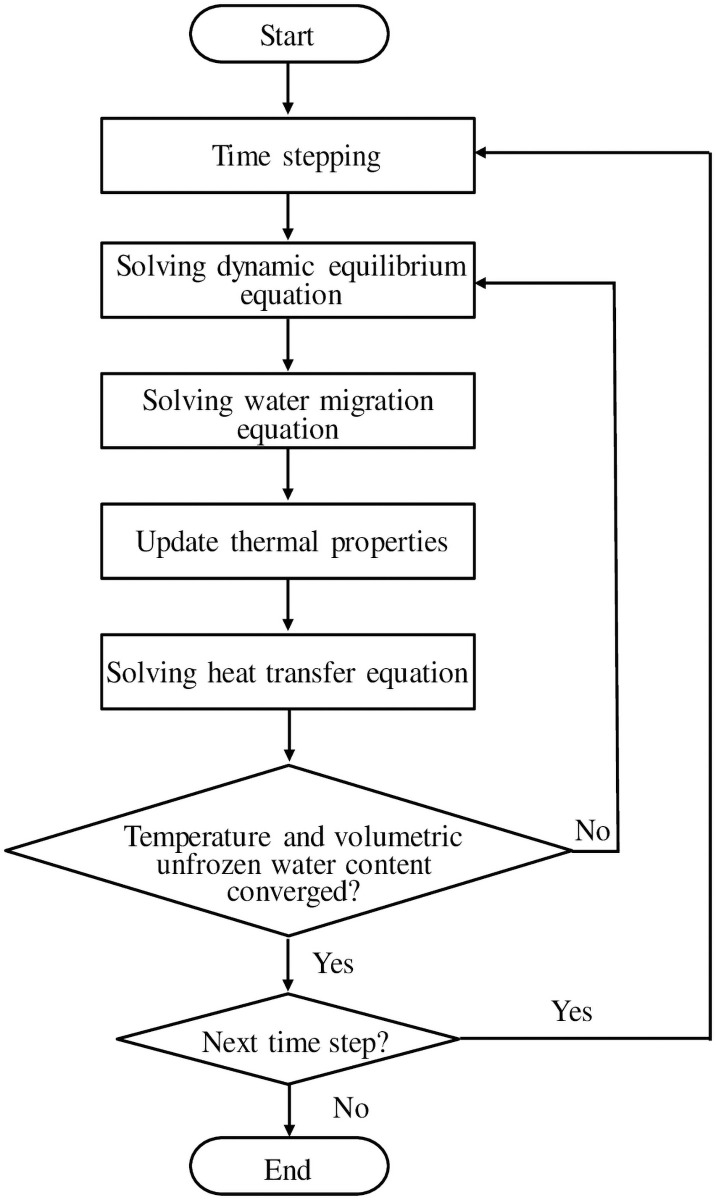
Flowchart of the computational process.

The general process of this approach is shown graphically in Fig 1.

### Finite volume method

All equations in this model have been spatially discretized using the finite volume method, which is widely used for numerical simulation of problems involving fluid flow and heat and mass transfer. As the finite volume method can be implemented on unstructured polygonal meshes, it is well-suited for modeling the hydrothermal process in frozen soil. In this study, the open-source C++ library OpenFOAM was selected for code execution, and the following parts describe the implementation of this solver.

All governing equations presented in Section 2 can be written in the general form:

$$\frac{\partial \rho \phi }{\partial t}=\nabla \cdot \Gamma \nabla \phi +S_{p}\phi +S_{c}$$ (18)

The parameters of interest are given in Table 1.

**Table 1:** Specific parameters of generalized variables used in numerical simulation.

Variable *ϕ*	*Ρ*	*Γ*	*S*_*p*_	*S*_*c*_
*T*	*C*	*λ*_*eff*_	0	*$L\rho_{i}\frac{\partial (r\theta_{u})}{\partial t}$*
*θ*_*u*_	*$1+\frac{\rho_{i}}{\rho_{w}}r$*	*D(θ*_*u*_*)*	*$\frac{\rho_{i}}{\rho_{w}}\frac{\text{d}r}{\text{d}t}$*	∇·*K*_*g*_(*θ*_*u*_)

**Fig 2:**
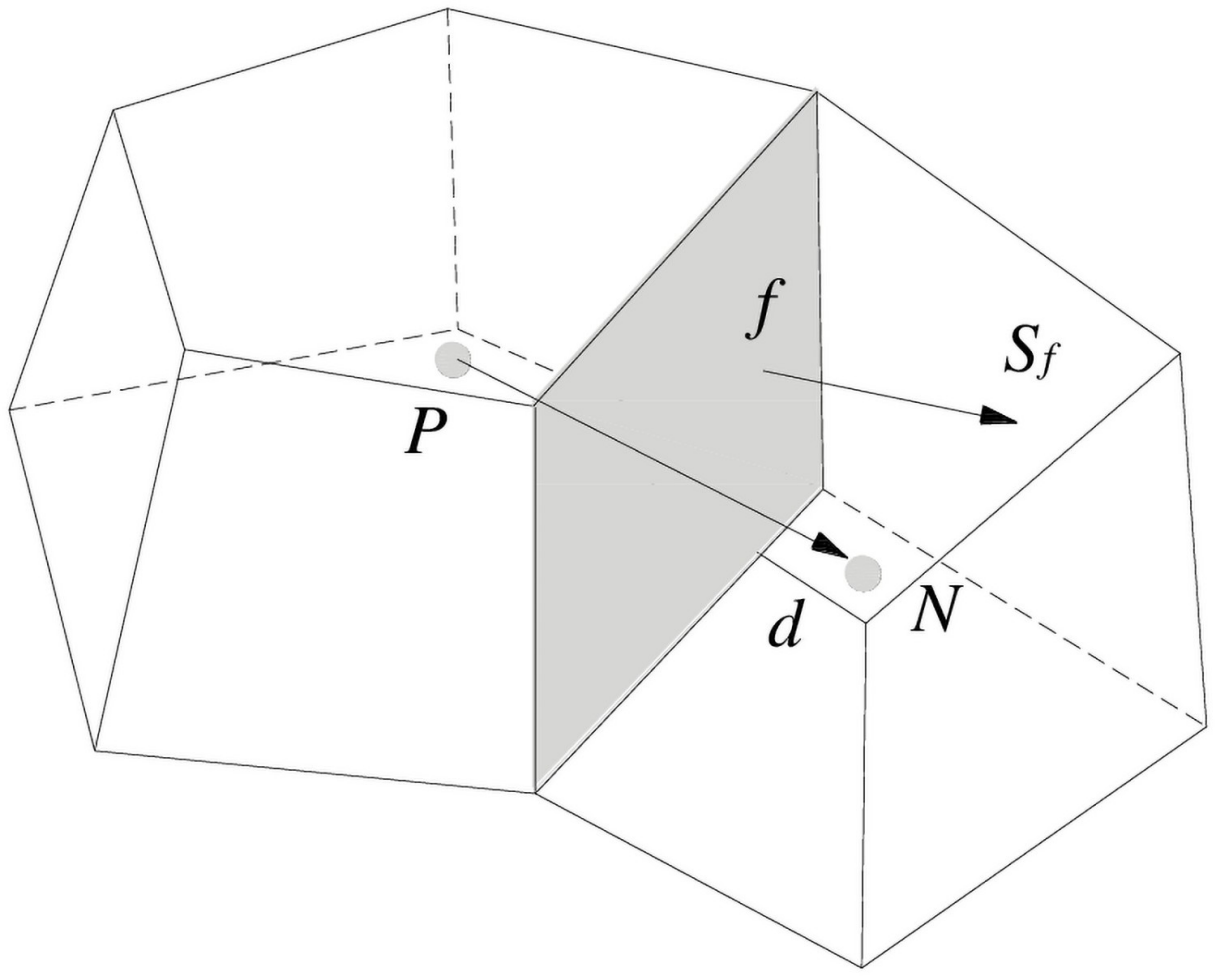
Parameters used in the finite volume discretization [27].

The first step of the discretization process is to partition the whole domain into non-overlapping elements, also known as finite volumes. The partial differential equations are then discretized into algebraic equations by integration over each discrete element. The parameters used in this discretization process are shown in Fig 2.

For example, consider two adjacent control volumes *P* and *N*, which share the plane *f*. Here, the vector *S*_*f*_ is the normal vector to *f* and the module is the area of *f*. By integrating Eq (18) over the control volume, Eq (18) can be transformed into

$$\int_{V}\frac{\partial \rho \phi }{\partial t}\text{d}V=\int_{V}\nabla \cdot \Gamma \nabla \phi \text{d}V+\int_{V}S_{p}\phi \text{d}V+\int_{V}S_{c}\text{d}V$$ (19)

Replacing the volume integrals of the Laplace terms by surface integrals based on the Gauss theorem, Eq (19) is thus transformed into

$$\int_{V}\frac{\partial \rho \phi }{\partial t}\text{d}V=\int_{\partial V}\Gamma \nabla \phi \text{d}S+\int_{V}S_{p}\phi \text{d}V+\int_{V}S_{c}\text{d}V$$ (20)

Replacing the surface integral by a summation over the control volume faces, the discretization equation on each cell can be described by

$$\frac{\partial \rho \phi }{\partial t}V=\sum_{f=1}^{N}\Gamma_{f}\nabla \phi S_{f}+S_{p}V\phi +S_{c}V$$ (21)

Then, the Laplacian terms in Eq (21) are discretized by the central difference scheme as described in Eq (22), which is always of second-order accuracy:

$$S_{f}\cdot {\left(\nabla \phi \right)}_{f}=|S_{f}|\frac{\phi_{N}-\phi_{P}}{\left|d\right|}$$ (22)

Calculation of the diffusion coefficient at the cell surface requires a conservative interpolation based on a harmonic mean, allowing the diffusion coefficients to guarantee continuity of both flux and current. For the two control volumes P and N shown in Fig 2, each of which has homogeneous diffusion coefficients (*Γ*_*P*_ and *Γ*_*N*_, respectively), the harmonic mean of the diffusion coefficient can be calculated by

$$\Gamma_{f}=\frac{\bar{PN}}{\frac{\bar{Pf}}{\Gamma_{P}}+\frac{\bar{fN}}{\Gamma_{N}}}$$ (23)

**Fig 3:**
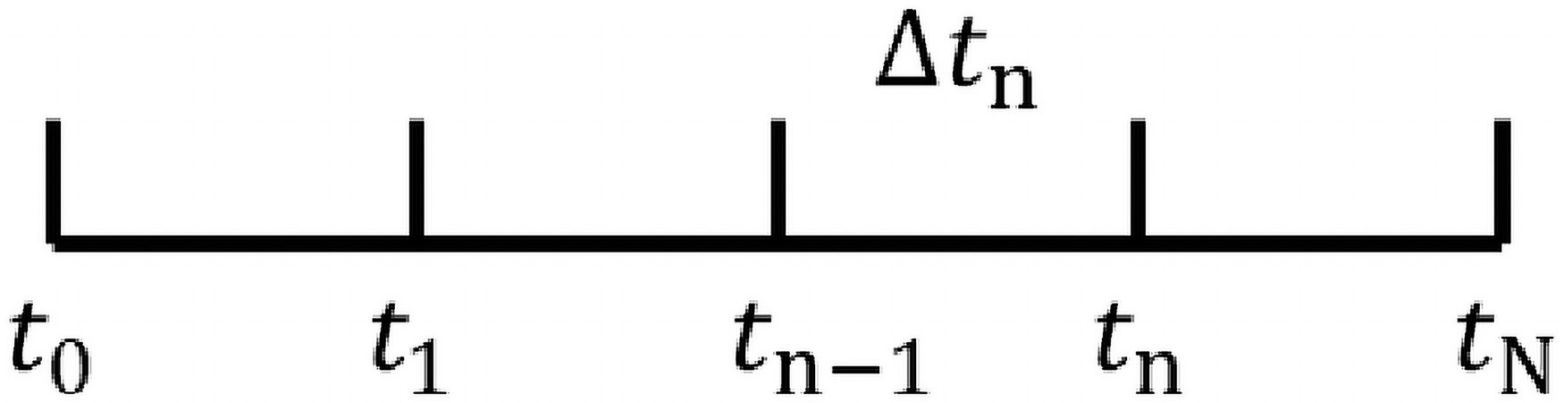
Parameters for the time discretization.

Eq (21) must be solved by a time step procedure. In addition, due to convergence and stability requirements in the calculation, a fully implicit backward difference scheme is used to discretize the time derivative term in Eq (21). The time variables are discretized, shown in Fig 3. This allows the discrete-time set and the corresponding series of time intervals [*t*_*n − 1*_, *t*_*n*_], n = 1, …, *N* to be derived, and the time step is:

$$\Delta t_{n}=t_{n}-t_{n-1}$$ (24)

The time derivative at time *t*_*n*_ can then be approximated by Eq (25), and all equations are solved step-by-step for each time interval.


$${\frac{\partial \rho \phi }{\partial t}|}_{t_{n}}=\frac{{\rho \phi |}_{t_{n}}-{\rho \phi |}_{t_{n-1}}}{△t_{n}}$$ (25)

Finally, the discretization of the above equations produces a set of algebraic equations that are solved to obtain discrete values.

## Validation and applications

### Validation

#### Experiment description

The theory and numerical solution presented in Sections 2 and 3 were validated against the results of one-dimensional soil freezing tests. The soil sample used in this experiment was collected from an embankment along the Baotou-Lanzhou Railway (BLR), North China, which is recognized as the first railway crossing a desert area of China. The BLR was constructed in the 1950s and has since suffered from severe frost damage due to the underlying natural surface soil, which was not well treated with respect to the frost issues. The soil utilized in our experiment was excavated at the BLR design milepost DK72+612.

Particle analysis of this soil showed the following composition: 0.74% fine gravel, 39.87% sand, 45.74% silt, and 13.65% clay. This soil also has a maximum dry density of 1.89 g/cm^3^, optimal moisture content of 13.0%, specific density of 2.74, liquid limit of 26.6%, and plastic limit of 13.4%. The sample was molded into a cylinder with 10 cm in height and 15 cm in diameter. Standard procedures for sample preparation were adhered to, such that the relative compaction was 90%, and the moisture content was 17.5%.

**Fig 4:**
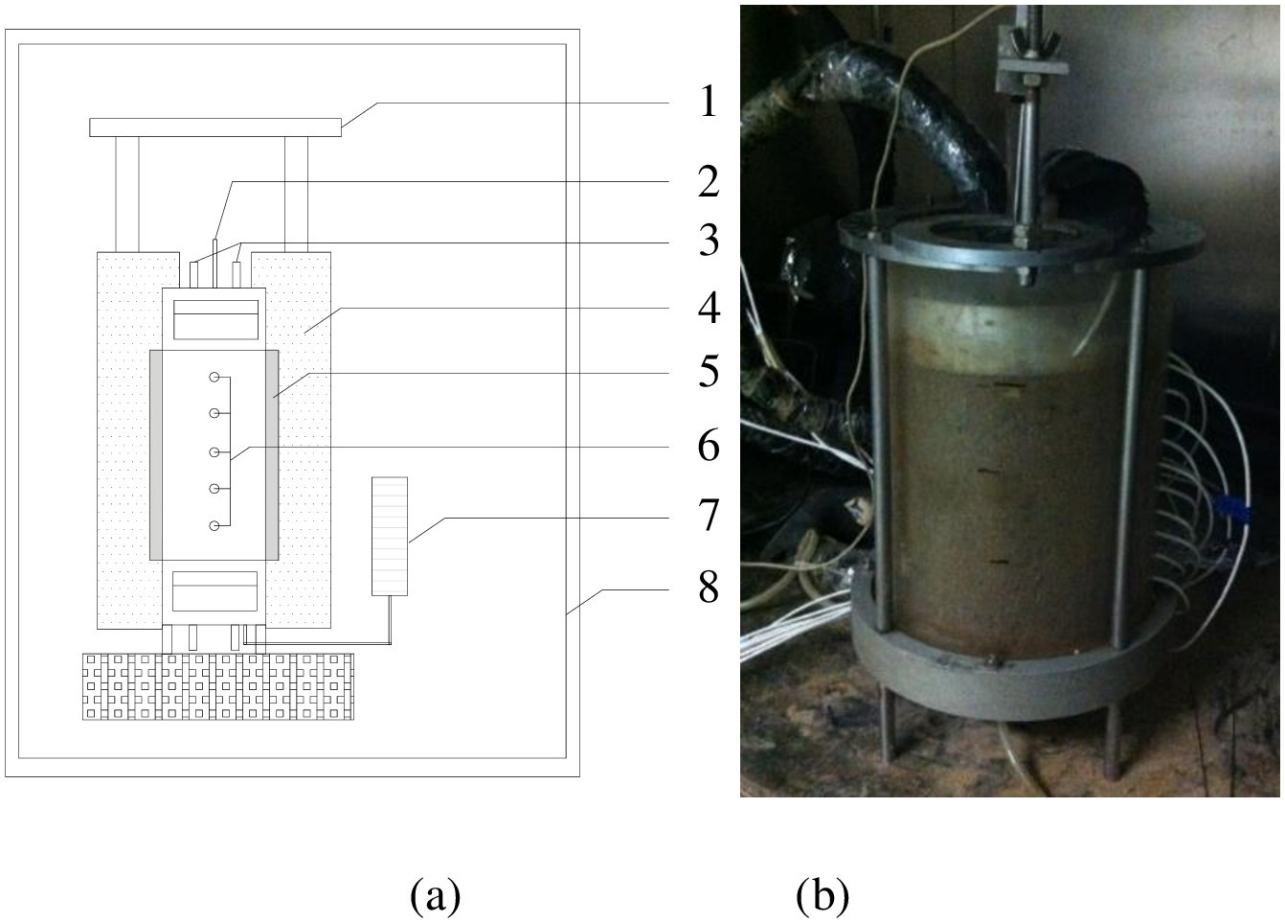
Experimental arrangement for soil freezing tests. (a) Apparatus and specimen profile and (b) image of the specimen: 1—Rigid frame; 2—displacement transducer; 3—inlet and outlet of cold bath; 4—insulating layer; 5—sample cell; 6—temperature probe; 7—Mariotte’s bottle; 8—temperature controlled chamber.

Freeze–thaw testing was conducted in the Frozen Soil Engineering Laboratory of Beijing Jiaotong University [28], as shown in Fig 4. The soil samples were conditioned by maintaining a constant temperature of +1°C before each test, which produced a uniform water content of 17.5%. During the experiment, the sides of the samples were thermally insulated and the temperature at the bottom of each soil column was held at +1°C. The top surfaces of the soil samples were exposed to a circulating fluid with a temperature of either −1.5°C or −3°C. This circulation kept the top surfaces of the soil samples at a constant temperature. After 48 h, the temperatures and moisture contents of the soils were measured as functions of the distances from the bottom of each column.

Numerical simulations of these experiments utilized the soil properties and water migration model parameters given in Tables 2 and 3. All simulations considered a mesh consisting of 50 control volumes with 0.2 cm spacing in the axial direction of the soil column. A fully implicit backward difference time-stepping routine was adopted with a time step of 30 s. The code implementation was deemed convergent if the liquid water contents and temperatures did not change by more than 1% between successive iterations at each time step for all test cases.

**Table 2:** Physical properties of the tested soil sample.

*C*_*s*_ (J∙m^−3^∙K^−^)	*C*_*i*_ (J∙m^−3^∙K^−^)	*C*_*u*_ (J∙m^−3^∙K^−^)	*λ*_*s*_ (W∙m^−1^∙K^−1^)	*λ*_*i*_ (W∙m^−1^∙K^−1^)	*λ*_*u*_ (W∙m^−1^∙K^−1^)
148.4	445.2	222.6	1.3	2.2	0.55

**Table 3:** Parameters of interest in the numerical simulations.

*a*_*0*_	*M*	*l*	*θ*_*r*_	*θ*_*s*_	*k*_*s*_ (m∙s^−1^)
2.65	0.26	0.5	0	0.19	5 ×10^−6^

In each simulation, the initial and boundary thermal conditions for the heat equation were specified as follows: *T (t* = 0 s) = 1°C and *T* (*t* > 0 s, *y* = 10 cm) = −0.75°C/−3°C, *T* (*t* > 0 s, *y* = 0) = 1°C. In addition, the initial and boundary conditions for the water equation were defined by: *θ*|_*t* = 0_ = 0.175, ${\frac{\partial \theta }{\partial n}|}_{t>0\;s,y\;=\;0.0}\;=\;0$, and ${\;\frac{\partial \theta }{\partial n}|}_{t>0\;s,y\;=\;10\;cm}\;=\;0$.

#### Comparison of experimental and numerical results

**Fig 5:**
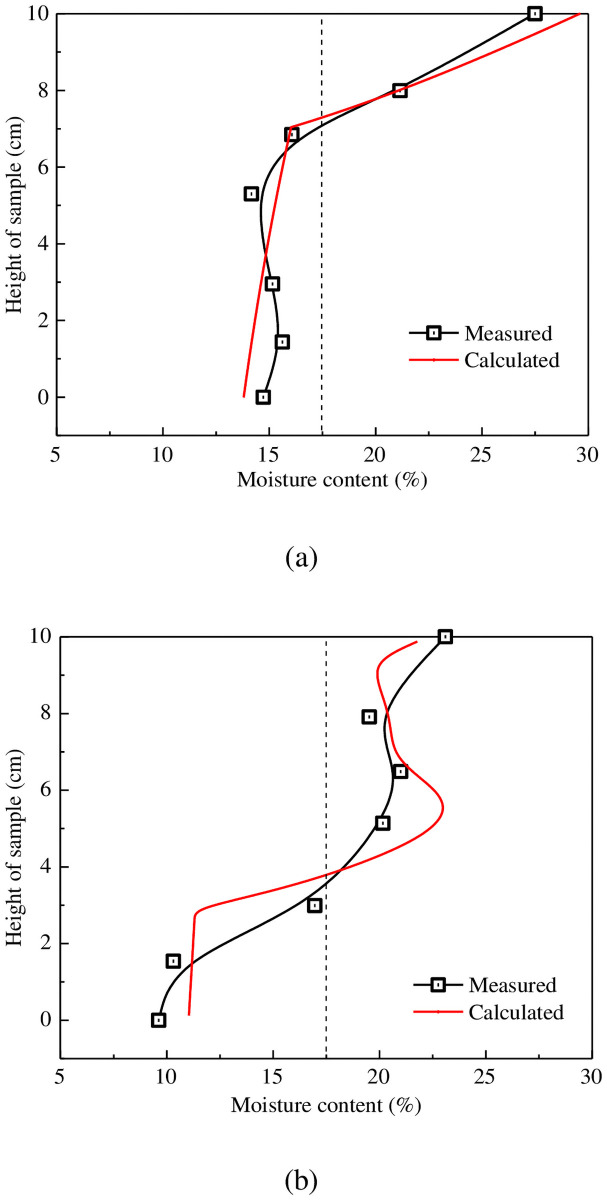
Distribution characteristics of soil moisture content after each freezing test for a soil-surface temperature of (a) −0.75°C and (b) −3°C.

The calculated total moisture content and vertical temperature distribution within the soil column were compared against the experimental results to verify the code program proposed. Fig 5 displays a comparison of datasets produced for different boundary thermal conditions, revealing that the calculated moisture contents show reasonable agreement with the measured values. Experiments conducted with a top temperature of −0.75°C formed a freezing front at the cold end (Fig 5a), and measured moisture contents in the adjacent unfrozen soil always exceeded those in other locations concerning the freezing front. Water diffusivity in the unfrozen zone thus drives fluid flow towards the cold face. Experiments using an upper temperature of −3.0°C (Fig 4b) produced a different moisture redistribution profile, whereby the measured moisture content was highest near to the cold end (top) of the column. Moving downwards through the column, the moisture content first decreases, then increases, and finally, the freezing point sharply decreases. Consequently, two peak moisture contents occur, one on the top surface of the soil sample and the second located at the position of the freezing edge. Formation of the latter can be explained by the freezing front moving relatively fast compared to diffusive fluid flow, such that the maximum velocity of water migration occurs at the freezing front, although further migration through the column is significantly reduced by the ice formation.

**Fig 6:**
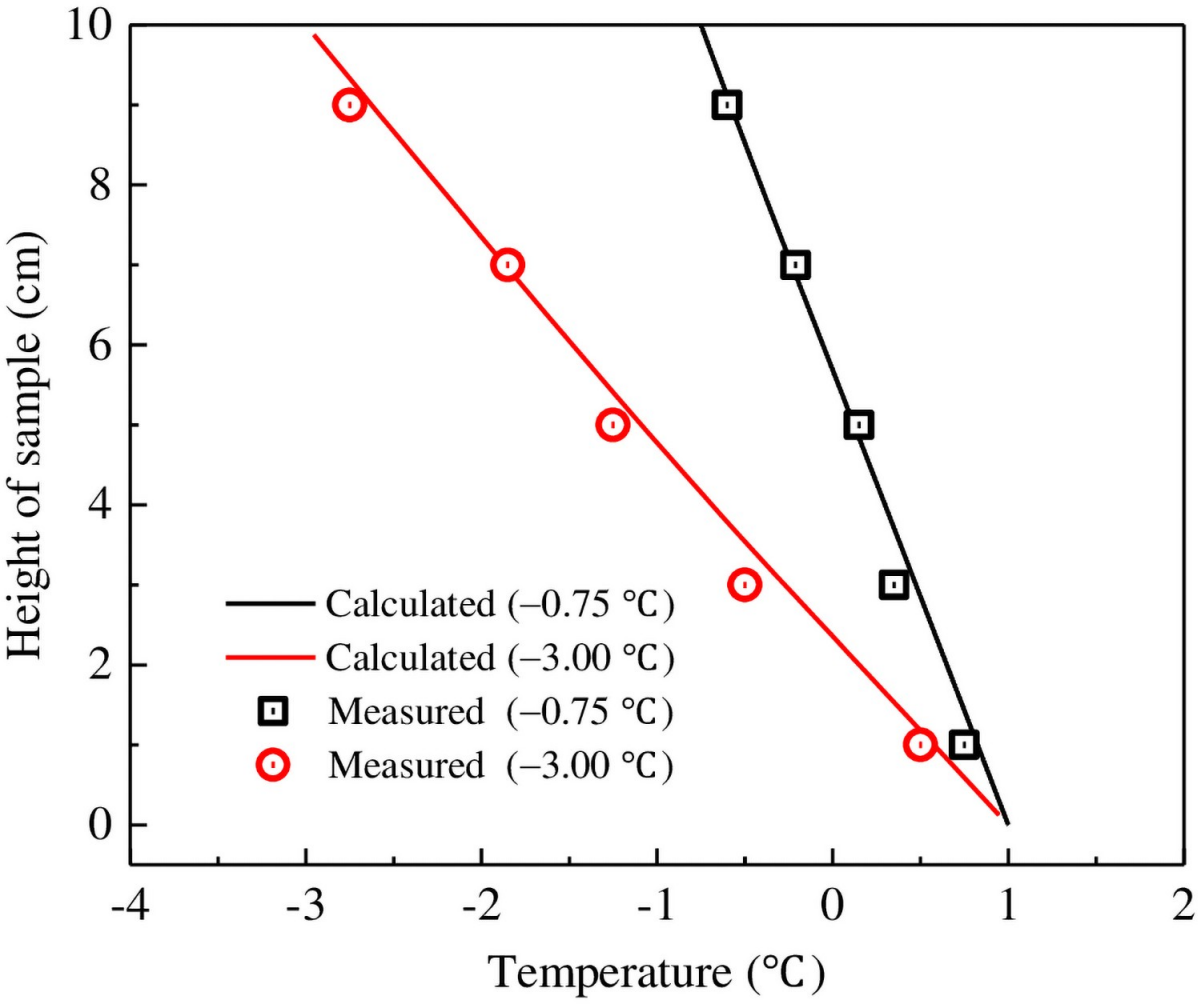
Temperature profiles within each soil sample after the freezing process.

These data also demonstrate a strong correlation between the measured and computed soil temperatures (Fig 6), both decrease linearly with depth in each experiment.

**Fig 7:**
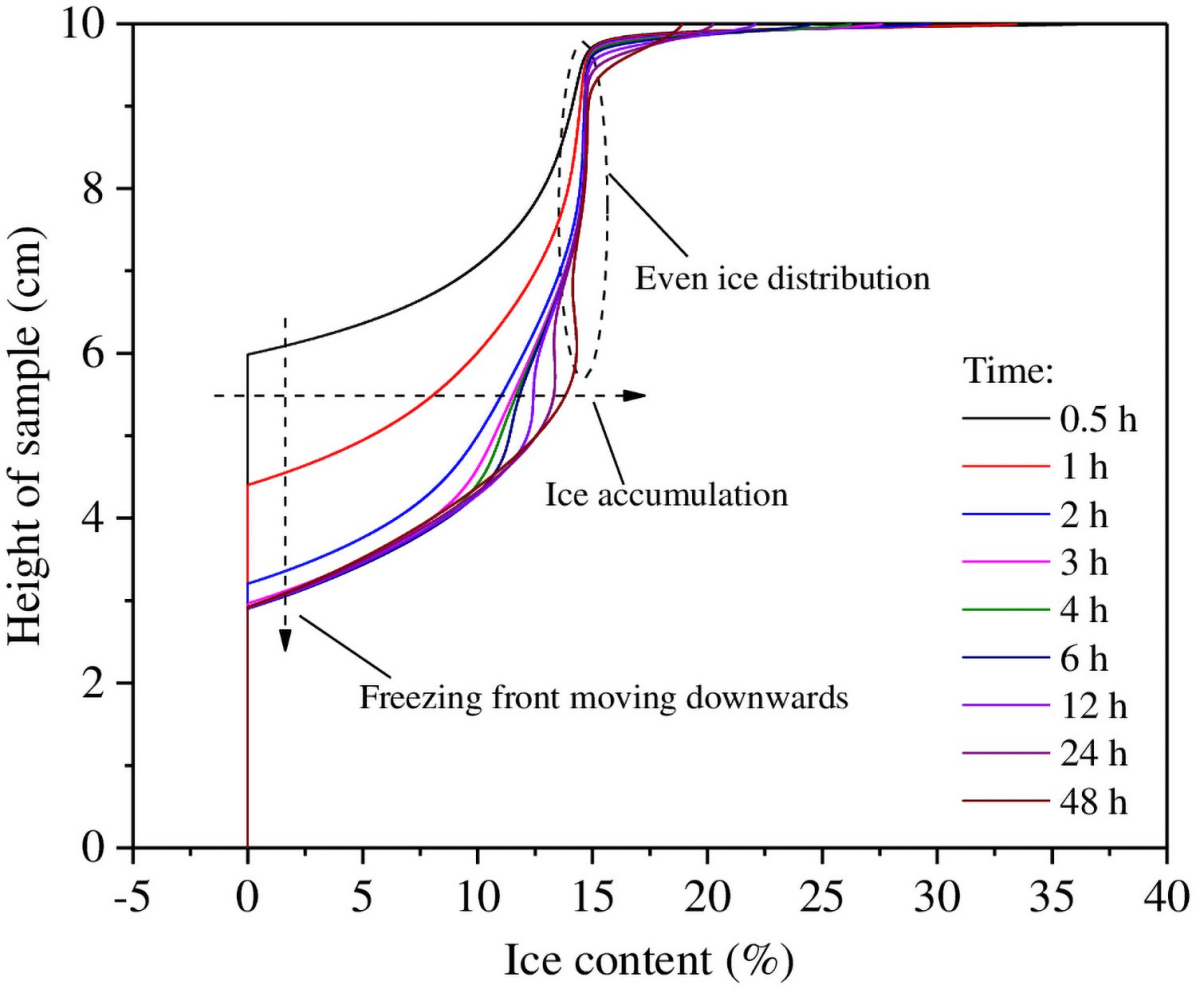
The calculated rate of ice accumulation for the experiment with a boundary temperature of −3°C.

Fig 7 presents calculated accumulation of ice content in the experiment using a soil-top temperature of −3°C. In this scenario, when the soil sample was subjected to a sub-freezing temperature, part of the pore water within the cold portion of the sample was the first to solidify into ice. Simultaneously, a gradient in water potential developed in the same direction as the temperature gradient. Water then diffused up from the warm portion to feed the accumulation of pore ice, resulting in water migration and an increase in ice content in the frozen area. At the same time, the liquid water content and the permeability of soil close to the freezing front both decreased. Therefore, it became increasingly difficult for water to flow to the frozen area behind the freezing front. With continued freezing, the freezing front advanced down the soil column to form a frozen area characterized by a relatively homogenous distribution of ice.

**Fig 8:**
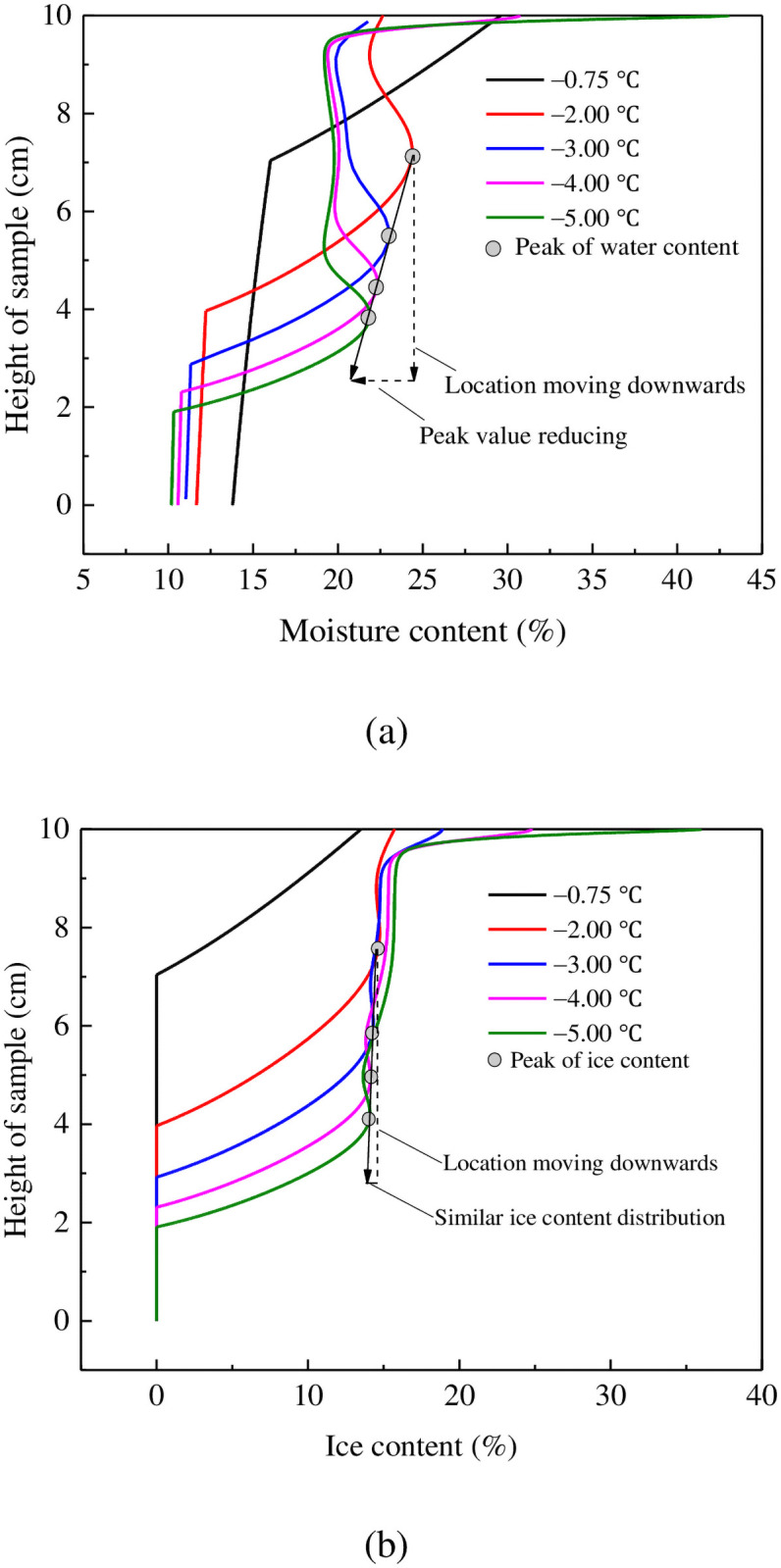
Calculated characteristics of moisture redistribution in soil samples with different soil-top temperatures: (a) volumetric moisture content; (b) volumetric ice content.

The distribution of water and ice within this soil column has also been predicted using our numerical model for boundary conditions ranging between −0.75°C and −5°C (Fig 8). These results all show an increase in total moisture content behind the advancing of freezing front, namely, a second peak in moisture content and the depletion in the unfrozen domain. The modeled speed at which the freezing front advances downwards through the column controls the moisture content increase in the frozen layer. For example, a slow-moving freezing front causes a larger increase in moisture content in the frozen soil (Fig 8a). In addition, the extent of the frozen area as a function of depth was modeled to increase as temperatures dropped, and the peak ice content also showed a slight reduction. However, compared with the degree of redistribution of moisture content at different temperatures, predicted ice contents had similar ranges. This indicates that the speed of the advancing freezing front primarily affects liquid water migration. These modeled results are reasonable and have been validated in the literature [29, 30]. The close correlation between observed (experimental) and calculated (simulated) results implies that our theoretical formulation can accurately describe the physical processes involved in freeze–thaw weathering.

### Applications

#### Railway embankment problem

**Fig 9:**
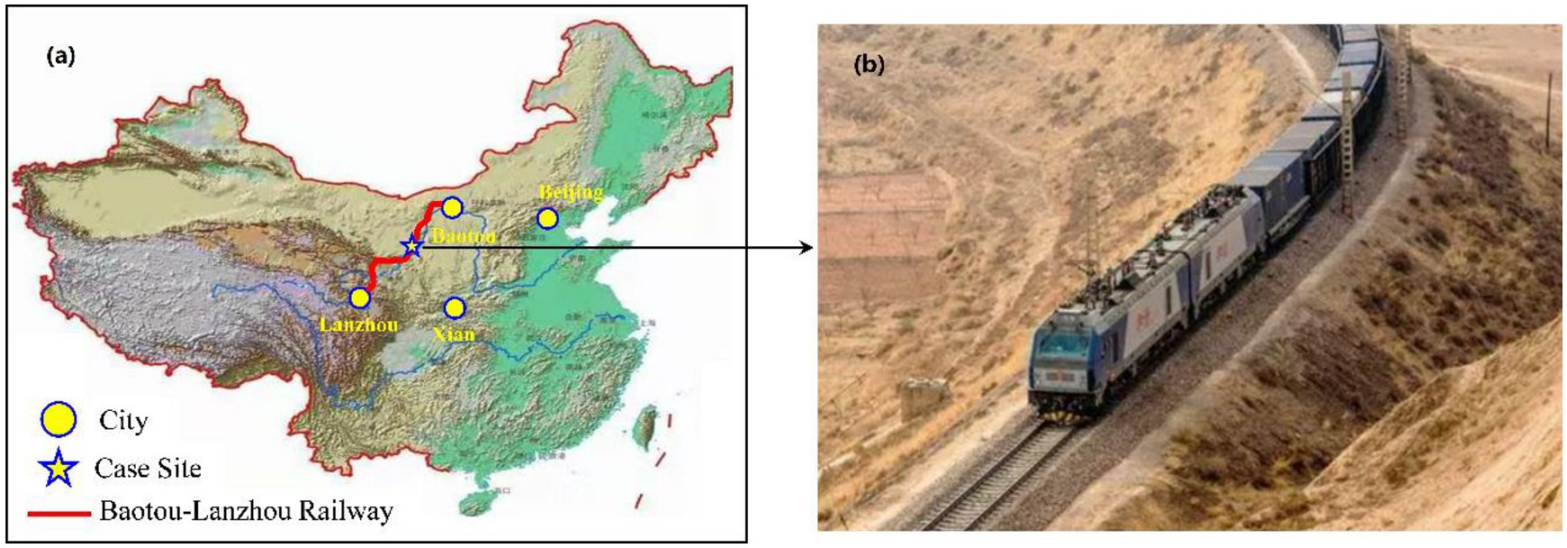
Trajectory of the Baotou-Lanzhou railway (a) and an overview of train running on this route (b).

The application of this model was illustrated by simulating the variation of temperature and moisture profiles in a railway embankment over a period. The site belongs to the Baotou-Lanzhou railway in the North China (Fig 9), where the maximum frost depth can reach 1–1.2 m below the natural ground. Field observations show that frost heave exceeds 20 mm in certain area, posing a threat to the safe operation of trains. The hydrothermal method was applied to develop a two-dimensional model of an embankment to evaluate the extent of frost heave defects and related long-term stability of the earth work.

#### Model and parameters

**Fig 10:**
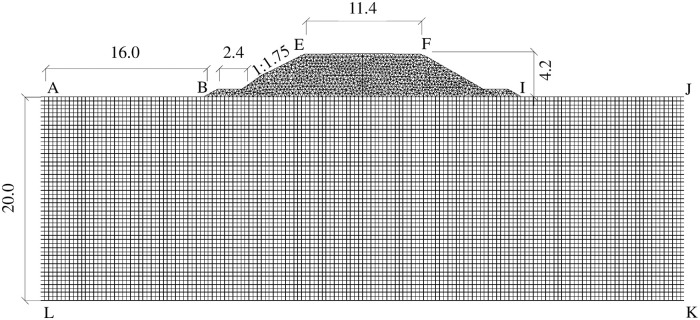
Mesh discretization of the railway embankment in modeling (Unit: m).

The spatial finite-volume meshes adopted for modeling of the railway embankment is shown in Fig 10. Here, the upper portion of the mesh is unstructured and the lower part is structured, simulating artificial and natural ground, respectively. The computational domain for both the embankment and natural ground used the soil parameters listed in Tables 2 and 3. This is a defensible assumption since the natural ground was once artificially compacted, and the embankment was constructed using the same soil without geotechnical pre-treatment or modification. The water content of the soil was set to 9%, based on the arid continental climate and deep-seated groundwater table in the region.

#### Boundary and initial conditions

In Fig 10, the upper boundary EF represents the top surface of embankment fills, and boundaries BE and FI are the embankment’s sloped surfaces. Horizontal boundaries AB and IJ represent the natural ground surfaces. Long-term observation of the test site allowed estimation of simplified boundary temperature conditions, as defined by Eq (26) and reported in Table 4.

$$T=T_{0}+A\text{sin}(\frac{2\pi }{p}t+\frac{\pi }{2})+R_{0}t$$ (26)

where *T*_*0*_ is the mean annual temperature, *A* is the annual amplitude of temperature variation, *p* is a vibration cycle equal to 8,760 h, *t* is the time in hours, and *R*_*0*_ is the heating rate, taken here to be 0.04°C/a.

**Table 4:** Thermal boundary conditions used in railway embankment modeling.

Model boundary	*T*_*0*_ (°C)	*A* (°C)
Natural ground surfaces (AB and IJ in Fig 10)	6.5	14.5
Slope surfaces of embankment fills (BE and FI in Fig 10)	6.5	14.5
Top surfaces of embankment fills (EF in Fig 10)	7.5	16.5

A constant temperature of *T* = 7°C was applied to the bottom surface (LK) of the computational model, which was situated 20 m below the natural ground surface. The left- and right-hand boundaries of the model were assumed to be adiabatic.

This numerical simulation assumed that the embankment was constructed on August 15, which typically records the highest annual temperature in this region. The initial temperature profile of the natural ground was obtained by calculating a 50-year transient solution for North China without considering long-term climate changes. The initial temperature distribution within the embankment fills was then determined by the top-surface temperature of natural ground at the point in time when the embankment was constructed. The spatiotemporal characteristics of the temperature and moisture fields within the embankment were then forward-modeled based on its construction 10 years later.

#### Results and analysis

**Fig 11:**
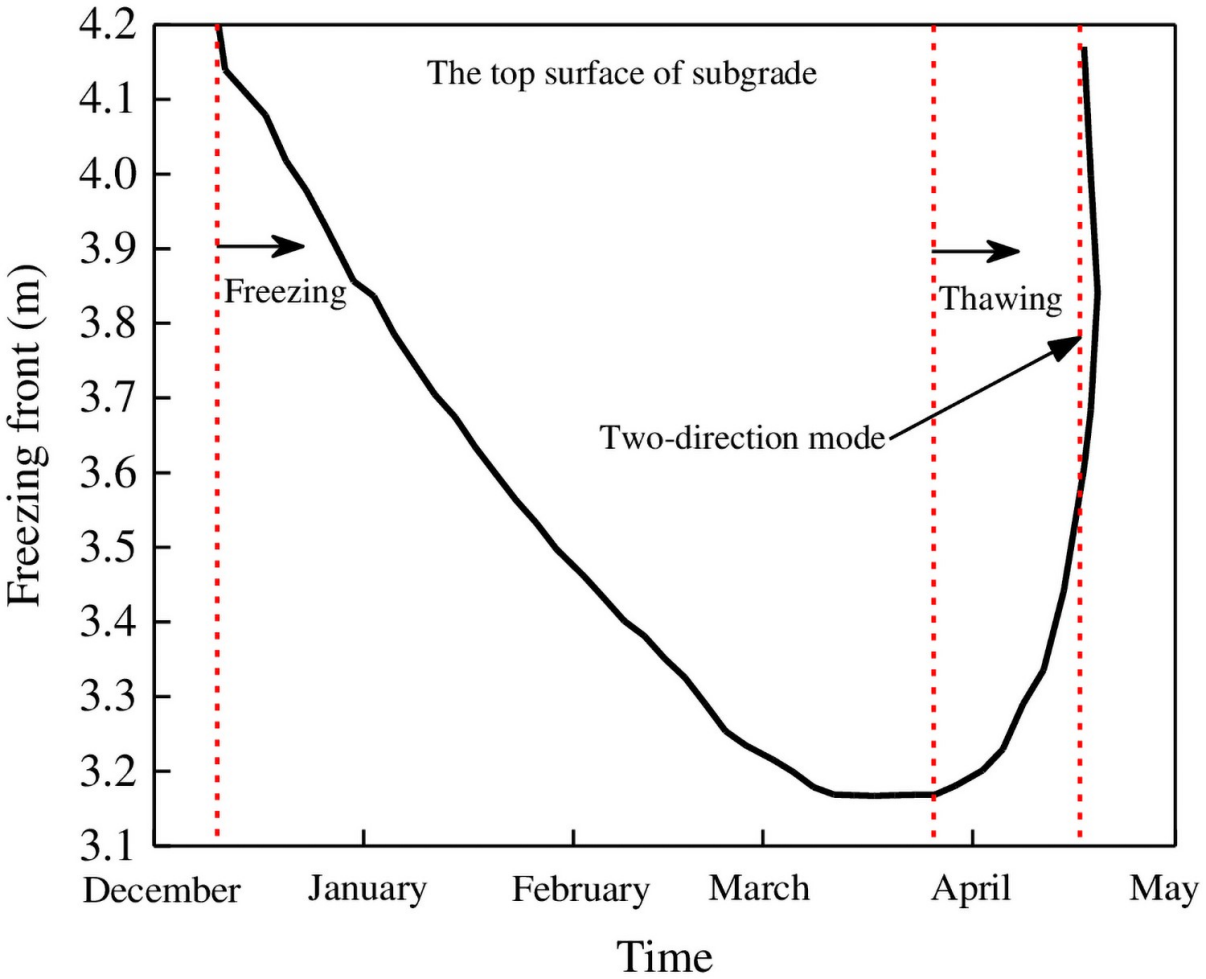
Calculated freezing depth beneath the centerline of the railway embankment section in the first year after construction.

Problems of frost heave in embankments are associated with the frost depth. For simplicity, 0°C was taken as the soil-freezing temperature in this simulation. The effects of the freeze–thaw process calculated by this model are shown in Fig 11. The upper layer of the embankment is predicted to freeze at the exposed surface on December 11 in the first year after construction, and the maximum freezing depth occurs at around March 27 of the following year. Interestingly, the subsequent thawing process simultaneously propagates from both the top and bottom of the embankment column central line, creating a two-direction mode (Fig 11). The predicted duration of this thawing period is relatively short, lasting approximately 20 days. By contrast, the predicted freezing period lasts from December of the first year to April of the next year, spanning almost 130 days.

**Fig 12:**
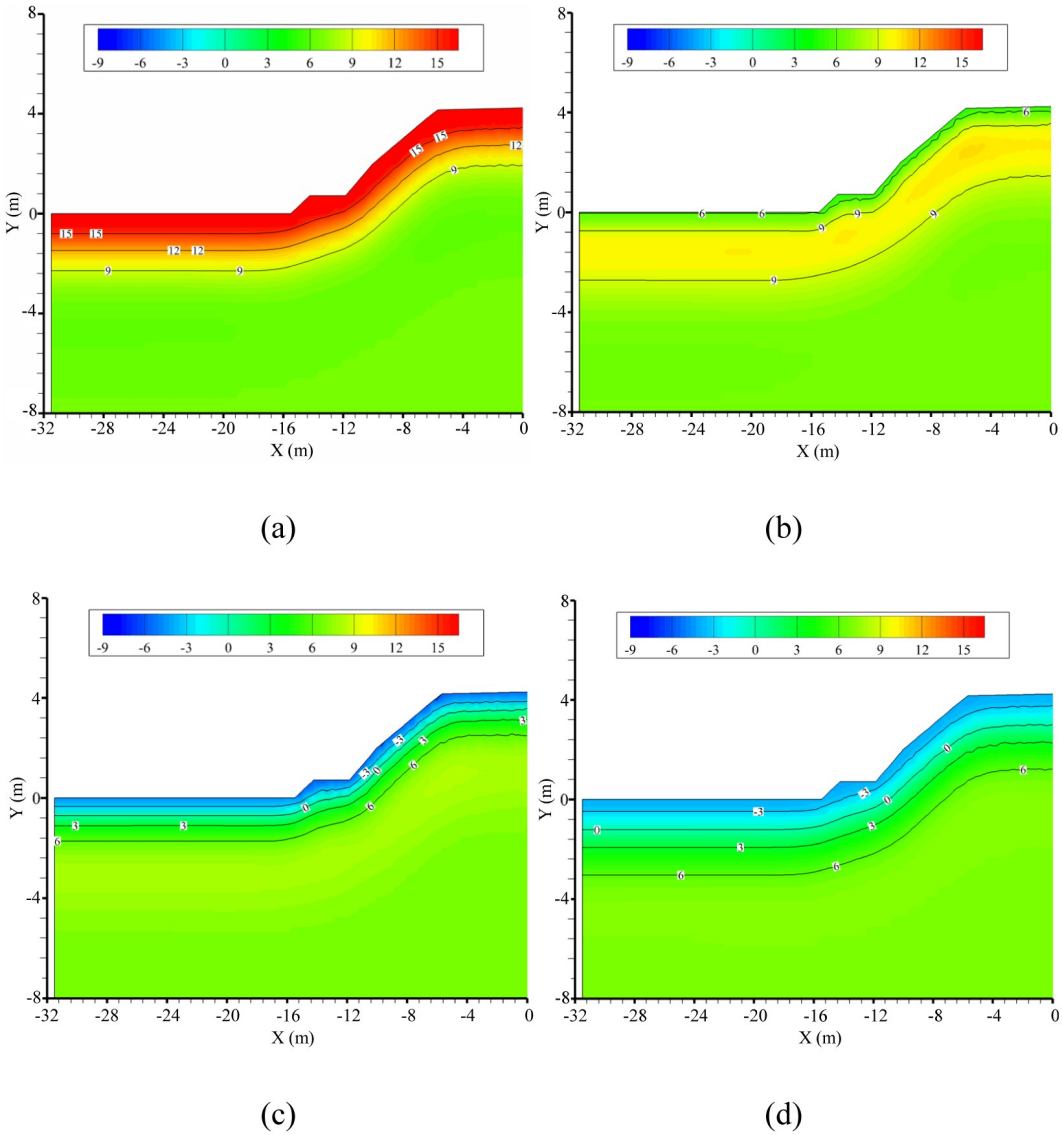
Modeled temperature (°C) distribution within the railway embankment in the 10^th^ year after construction on (a) October 1, (b) December 1, (c) February 1, and (d) April 1.

Fig 12 shows the temperature distribution of the embankment in the 10^th^ year after construction. During annual cooling associated with decreasing air temperature, the cooling zone of the embankment section is enlarged continuously. The freezing depth on April 1 is situated −1.16 m below the natural ground surface, while the freezing depth of the embankment under the centerline is 1.29 m and 0.13 m thicker than that under the natural ground surface. Moreover, the minimum temperature occurs on the shoulders of the embankment, which suffers from a two-way invasion of cold fronts, causing them to experience the greatest freezing depth. This result implies that preventative measures should be adopted to avoid asymmetrical frost heave and crack formation on embankments, which can otherwise severely affect the stability of railway foundations.

**Fig 13:**
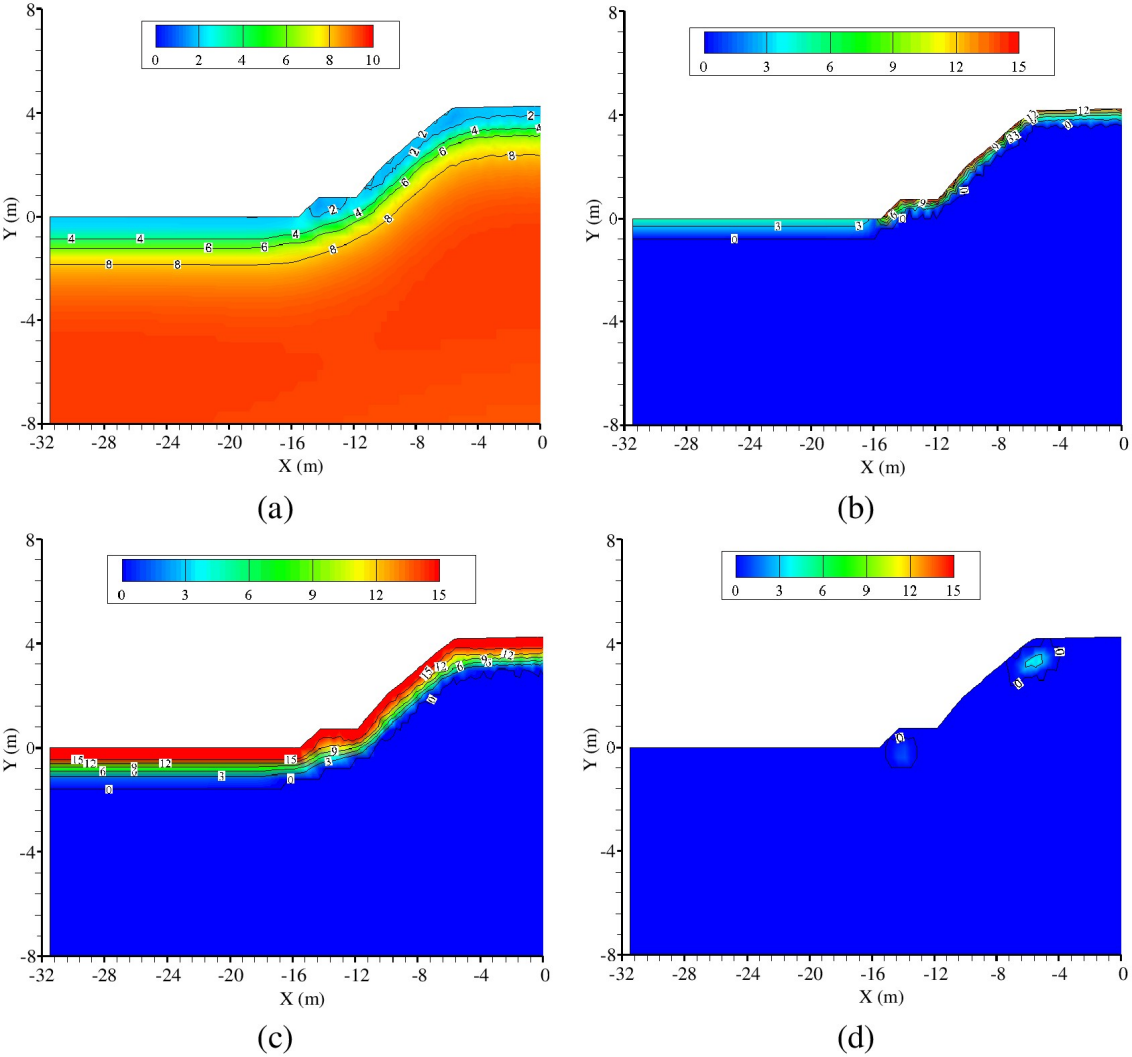
Calculated volumetric moisture content (%) contours ten years after construction: Unfrozen water content on December 1 (a); ice content on December 1 (b), February 1 (c), and April 1(d).

Spatiotemporal moisture redistribution is an important parameter for evaluating the long-term stability of railway embankments. Fig 13 shows the distribution of volumetric moisture content predicted during the freezing period defined above. In the early freezing period, unfrozen water in the shallow layers of the embankment decreases in volume, while free water within the foundation migrates upward towards the frozen fringe (Fig 13a). As a result, moisture accumulated in the frozen fringe, and a layer of ice gradually formed (Fig 13b and 13c). Thereafter, a frozen portion of the modeled soil core appeared beneath the ground surface due to initiation of a two-direction thawing mode (Fig 13d), which shrunk over time. The modeled ice content near to the top and shoulder of the railway embankment increased significantly during this period, although was not predicted to be abundant at the foot of the embankment due to the protective effect of the revetment. Hence, the construction of a revetment is suggested to be a cost-effective measure for preventing frost accumulation, and although additional precautionary measures should be adopted at other locations on the surface of the embankment to prevent ice formation. Critically, the spatiotemporal effect of frost heave within the subgrade can be predicted via our numerical code, purely based on the calculated temperature profile and ice content distribution.

**Fig 14:**
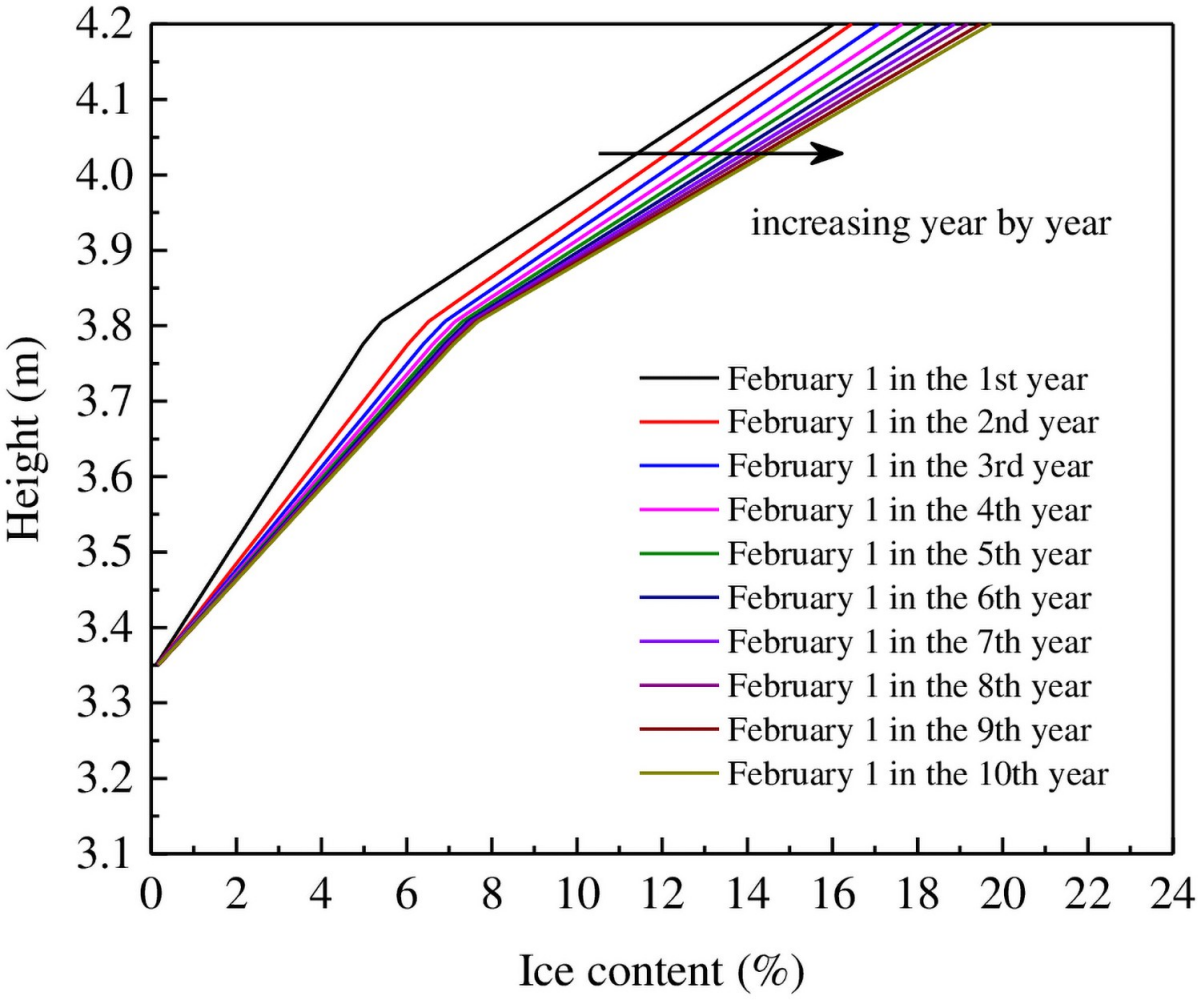
Modeled variations of ice content (%) under the centerline of the railway embankment section on February 1 each year after construction.

Fig 14 shows how volumetric ice content is predicted to vary directly beneath the centerline of the railway embankment section for 10 years after its construction. These data reveal that ice should accumulate progressively closer to the upper part of the embankment over time, suggesting that moisture migration inside the embankment should also continue on an annual basis due to annual temperature changes. As such, moisture-blocking layers should be installed within railway embankments to ensure their long-term stability.

## Conclusions

In this paper, a numerical model was presented for the hydrothermal process in unsaturated soils upon freezing and thawing. The model was developed using the open-access OpenFOAM platform. Numerical simulations of laboratory freezing experiments show close correlations between predicted and measured values for spatiotemporal changes in moisture content and temperature, indicating the robustness of our code. Following this validation, this model was applied to predict how temperature and moisture redistribution varies in a railway embankment over time, which are important physical parameters that govern its long-term stability. Our preliminary results demonstrate that the numerical model and associated code presented herein may be useful for design, maintenance, and research on other embankments in seasonally frozen regions.

The data and results presented in this study confirm that our model effectively considers multiple aspects of temperature and fluid flow, and successfully resolves the interdependence between heat and moisture transfer in soil. Future research projects will quantify the magnitude of deformation of soil columns due to freeze–thaw cycles, and field-based data will be collected and integrated into our code to better describe the hydraulic and thermal properties of unsaturated soil.

## Supporting information

S1 Data Data for railway embankment simulation. (ZIP)

S2 Data Data for soil column simulation. (ZIP)
